# T315I mutation of BCR-ABL1 into human Philadelphia chromosome-positive leukemia cell lines by homologous recombination using the CRISPR/Cas9 system

**DOI:** 10.1038/s41598-018-27767-6

**Published:** 2018-07-02

**Authors:** Minori Tamai, Takeshi Inukai, Satoru Kojika, Masako Abe, Keiko Kagami, Daisuke Harama, Tamao Shinohara, Atsushi Watanabe, Hiroko Oshiro, Koshi Akahane, Kumiko Goi, Eiji Sugihara, Shinichiro Nakada, Kanji Sugita

**Affiliations:** 1 Department of Pediatrics, School of Medicine, University of Yamanashi, Chuo, Japan; 2 Innovation Medical Research Institute, Research and Development Center for Precision Medicine, University of Tsukuba, Ibaraki, Japan; 3 Department of Bioregulation and Cellular Response, Graduate School of Medicine, Osaka University, Osaka, Japan

## Abstract

In many cancers, somatic mutations confer tumorigenesis and drug-resistance. The recently established clustered regularly interspaced short palindromic repeats (CRISPR)/Cas9 system is a potentially elegant approach to functionally evaluate mutations in cancers. To reproduce mutations by homologous recombination (HR), the HR pathway must be functional, but DNA damage repair is frequently impaired in cancers. Imatinib is a tyrosine kinase inhibitor for BCR-ABL1 in Philadelphia chromosome-positive (Ph+) leukemia, and development of resistance due to kinase domain mutation is an important issue. We attempted to introduce the T315I gatekeeper mutation into three Ph+ myeloid leukemia cell lines with a seemingly functional HR pathway due to resistance to the inhibitor for poly (ADP) ribose polymerase1. Imatinib-resistant sublines were efficiently developed by the CRISPR/Cas9 system after short-term selection with imatinib; resulting sublines acquired the T315I mutation after HR. Thus, the usefulness of CRISPR/Cas9 system for functional analysis of somatic mutations in cancers was demonstrated.

## Introduction

Imatinib is a tyrosine kinase inhibitor (TKI) against BCR-ABL1 fusion tyrosine kinase derived from Philadelphia chromosome in chronic myeloid leukemia (CML) and Philadelphia chromosome-positive acute lymphoblastic leukemia (Ph+ ALL)^1,2^. Imatinib can achieve durable cytogenetic and molecular remissions not only in CML patient^3^ but also in patients with Ph+ ALL in combination with conventional chemotherapy^4,5^. Despite the remarkable success of imatinib, resistance has been identified due to point mutations in the *BCR-ABL1* kinase domain^2,6,7^. Among these mutations, the T315I gatekeeper mutation confers resistance to both imatinib^6,8^ and second-generation TKIs such as nilotinib and dasatinib^9^. Finally, ponatinib was developed as a potent TKI that can inhibit all critical kinase domain mutations including T315I^10^.

To investigate the biological significance of T315I mutation and to develop the therapeutic strategy overcoming TKI-resistance, a line of cellular models of T315I-positive leukemia was established. The most common system was murine IL-3-dependent Baf3 cells expressing *BCR-ABL1* or its mutant cDNAs that were transduced with retrovirus vector^8,11–13^. BCR-ABL1 and its mutants induced spontaneous cell growth of Baf3 in the absence of IL-3. The other commonly used system was imatinib-resistant sublines of human Ph+ leukemia cell lines. A couple of imatinib-resistant sublines with T315I mutation were established after long-term culture of imatinib-sensitive Ph+ leukemic cell lines in the presence of increasing concentrations of imatinib^14–17^. However, it has also been reported that long-term culture with increasing concentrations of imatinib induced imatinib resistance due to amplification of the *BCR-ABL1* fusion gene and overexpression of P-glycoprotein (P-gp)^18,19^. This suggests that imatinib-resistant sublines with T315I (established after long-term selection with imatinib) may acquire additional mechanisms for imatinib resistance. Thus, to directly test the effect of the T315I mutation, establishing a new system that enables the T315I mutation to be introduced into imatinib-sensitive Ph+ leukemia cell lines without long-term imatinib selection is desirable.

The clustered regularly interspaced short palindromic repeats (CRISPR)/Cas9 system consists of a Cas9 endonuclease and a single-guide RNA (sgRNA) that allows sequence-specific gene editing in mammalian cells^20–22^. CRISPR/Cas9 effectively introduces target double-stranded brakes (DSBs) by recognizing a NGG 3-base-pair protospacer adjacent motif (PAM) and causing hybridization between the 20-nucleotide stretch of the sgRNA and the DNA target site, which triggers Cas9 to cleave both DNA strands. DSBs activate two intrinsic repair mechanisms: non-homologous end-joining (NHEJ) and homologous recombination (HR). NHEJ (the predominant pathway for repair of DSBs) can introduce unpredictable insertions and deletions (indels) resulting in knockout alleles through the introduction of frame-shift mutations. HR is achieved in the presence of a single-stranded oligodeoxynucleotides (ssODN) template homologous to the sequences flanking the cleavage site. HR using the CRISPR/Cas9 system could be useful for introducing the T315I mutation into human Ph+ leukemia cell lines; however, to our knowledge, no reports have described success in purely introducing the point mutation of endogenous gene into human leukemia cells by HR using the CRISPR/Cas9 system.

To introduce HR-mediated gene editing with the CRISPR/Cas9 system in leukemia cells, the intrinsic HR pathway of leukemia cells must be functionally active. Most cancer cells demonstrate increased genomic instability due to impairment in repair pathways for DNA damage^23^. This seems to be true in Ph+ leukemia cells^24^. Although inactivating mutations in the HR pathway has been rare in leukemia^25^, BCR-ABL1 reportedly represses genes involved in the HR pathway such as *BRCA1* and *RAD51*^24–28^. These observations suggest that DSBs by the CRISPR/Cas9 system might fail to induce HR-mediated gene editing in Ph+ leukemia cells. Poly (ADP) ribose polymerase1 (PARP1) repairs DNA single-stranded breaks (SSBs). Inhibitors for PARP1 have recently been identified and are promising agents for cancer treatment^23,24,29-32^. PARP1 inhibitors generate DSBs during the replication process by trapping the inactivated PARP1 onto the SSB and/or disrupting the DNA repairment. Subsequently, PARP1 inhibitors induce apoptosis of cancer cells that have deficiency in key components of the HR pathway, which utilizes the sister chromatid as a template for a correct repairment of the DNA sequence. In contrast, cancer cells with intact HR pathways repair DSBs and show resistance to PARP1 inhibitors. Thus, sensitivities of cancer cells to PARP1 inhibitors might be useful to evaluate their status of the HR pathway.

In the present study, using the CRISPR/Cas9 system and ssODN, we attempted to introduce the T315I mutation into three Ph+ myeloid leukemia cell lines that showed resistance to olaparib (a PARP1 inhibitor). After short-term imatinib selection, we obtained imatinib-resistant sublines and confirmed that imatinib-resistant sublines successfully acquired T315I mutation of *BCR-ABL1* as a result of HR-mediated gene editing.

## Results

### Ph+ myeloid leukemia cell lines showed resistance to PARP1 inhibitor

**Figure 1:**
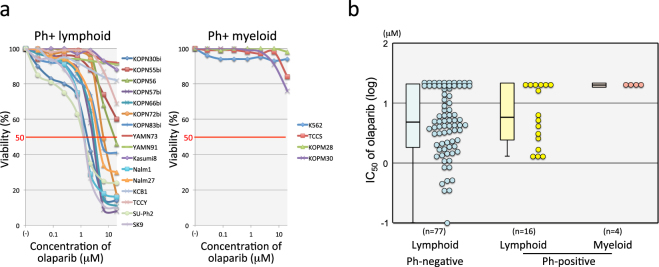
Sensitivity of Ph+ leukemia cell lines to PARP1 inhibitor. (**a**) Dose-response curve of olaparib sensitivity in Ph+ leukemia cell lines. The vertical axis indicates the percentage (%) viability in the alamarBlue cell viability assay and the horizontal axis indicates log concentration of olaparib (μM). Left and right panels indicate the sensitivities of Ph+ lymphoid and Ph+ myeloid cell lines, respectively. (**b**) Olaparib sensitivity in 16 Ph+ lymphoid, 4 Ph+ myeloid, and 77 Ph-negative lymphoid leukemia cell lines. The vertical axis indicates the IC_50_ value of olaparib. The IC_50_ value of cell lines in each group is also represented by a boxplot.

To introduce a T315I mutation in Ph+ leukemia cell lines by HR-mediated gene editing with the CRISPR/Cas9 system, the endogenous HR pathway must be functionally active. However, previous reports demonstrated that BCR-ABL1 represses genes involved in the HR pathway such as *BRCA1* and *RAD51*^24,26–28^. To select Ph+ leukemia cell lines with functional HR pathway, we evaluated the sensitivity to olaparib (one of PARP1 inhibitors)^32^. We determined the IC_50_ values of olaparib in 20 Ph+ (16 lymphoid and 4 myeloid) leukemia cell lines in comparison with 77 Ph-negative (Ph−) B-cell precursor ALL (BCP-ALL) cell lines using the alamarBlue cell viability assay (Table 1 and Fig. 1a). Distributions of mean IC_50_ values of three independent assays were similar between 16 Ph+ and 77 Ph− lymphoid leukemia cell lines (Fig. 1b). Median IC_50_ was 5.8 μM and 4.7 μM in Ph+ and Ph− lymphoid leukemia cell lines, respectively. Of note, all Ph+ myeloid leukemia cell lines were highly resistant to olaparib (Fig. 1a,b), suggesting that the HR pathway is functionally active in these Ph+ myeloid leukemia cell lines.

**Table 1:** Characterization and olaparib sensitivity of Ph+ leukemia cell lines.

Cell line	Type	BCR-ABL1	IC50 of olaparib (μM)	
			Median	Range
**Myeloid**				
K562	CML-BC	p210	>20	>20
TCCS	CML-BC	p210	>20	>20
KOPM28	CML-BC	p210	>20	>20
KOPM30	Ph+ AML	p190	>20	>20
**Lymphoid**				
KOPN30bi	Ph+ ALL	p190	1.7	1.2–2.1
KOPN55bi	CML-BC	p210	>20	>20
KOPN56	Ph+ ALL	p210	16	3.4->20
KOPN57bi	Ph+ ALL	p190	2.6	1.1–3
KOPN66bi	Ph+ ALL	p190	3	2.3–3.5
KOPN72bi	Ph+ ALL	p190	6.4	3.4–9.4
KOPN83bi	Ph+ ALL	p190	5.2	4.4- > 20
YAMN73	Ph+ ALL	p203	>20	>20
YAMN91	Ph+ ALL	p190	>20	>20
Kasumi8	Ph+ ALL	p190	>20	>20
Nalm1	CML-BC	p210	1.3	0.3–1.5
Nalm27	Ph+ ALL	p210	4.1	4–4.4
KCB1	Ph+ ALL	p190	>20	>20
TCCY	Ph+ ALL	p210	>20	>20
SU-Ph2	Ph+ ALL	p190	1.3	0.57–2.6
SK9	Ph+ ALL	p190	1.3	1–1.6

### Introduction of T315I mutation using the CRISPR/Cas9 system

**Figure 2:**
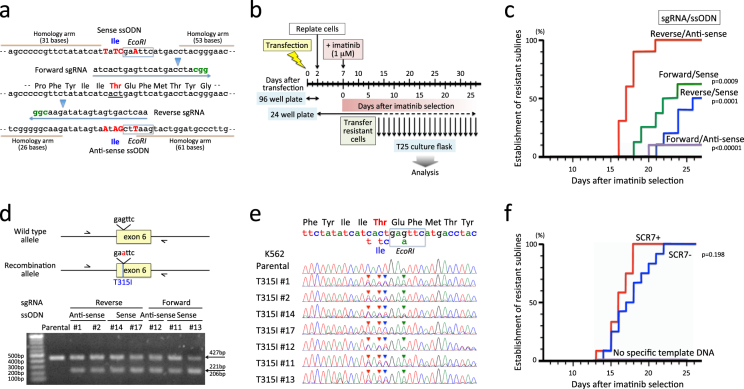
Introduction of the T315I mutation in K562. (**a**) Schematic diagram of the CRISPR/Cas9 system. Wild-type genomic sequence is indicated at the middle of panel, and codon 315 is underlined. Two (forward and reverse) sgRNA sequences and two (sense and anti-sense) complementary template ssODN sequences are indicated above and below of the wild type sequence. PAM motifs are indicated in green. Arrowheads indicate cleavage sites. In the template showing ssODN sequences, four mutated nucleotides are capitalized in red. Blue boxes indicate EcoRI sites. (**b**) Experimental workflow. sgRNA was electroplated in combination with recombinant Cas9 protein and ssODN. Forty-eight hours after transfection, the cells were plated into 10–12 wells of a 24-well plate. Five days latter, the cells were cultured in the presence of 1 μM of imatinib, and expanded imatinb-resistant cells were transferred to culture flask and expanded in the absence of imatinib. (**c**) Efficiencies in establishment of imatinib-resistant sublines. The vertical axis indicates percentage (%) establishment of imatinib-resistant sublines, and the horizontal axis indicates days after imatinib selection. Kaplan-Meier analysis of efficiency was performed between the combination of reverse sgRNA and anti-sense ssODN and the other three combinations. Combinations of sgRNA and ssODN are indicated with p-values in the Kaplan-Meier analysis. (**d**) PCR analysis of acquired mutation by EcoRI digest. In the upper panel, arrows indicate primers for PCR amplification of genomic DNA. The lower panel demonstrates electrophoresis of PCR products after EcoRI digestion. Combinations of sgRNA and ssODN are indicated above the panel. Full-length gel is presented in Supplementary Fig. S1. (**e**) Sequences of PCR products. Wild-type genomic and amino acid sequences and mutations in template sequence for HR are indicated at the top of the panel. Arrowheads in each sequence indicate mutations as a result of HR. (**f**) Effect of SCR7 treatment. The vertical axis indicates % establishment of resistant sublines, and the horizontal axis indicates days after imatinib-selection. Efficiencies in SCR7-treated cells (red) and untreated cells (blue) electroporated with reverse sgRNA and anti-sense ssODN are indicated by p-value in the Kaplan-Meier analysis. No imatinib-resistant sublines were obtained in the SCR7-treated cells electroporated with carrier ssODN instead of specific template ssODN.

To introduce T315I mutation by HR-mediated gene editing with the CRISPR/Cas9 system, we prepared two CRISPR guide sequences close to the target site (Fig. 2a). Forward sgRNA binds downstream of the target site in the coding strand direction, while reverse sgRNA binds upstream of the target site in the non-coding strand direction. Off-target hit scores of forward and reverse sgRNAs were 70 and 55, respectively, using a CRISPR design tool (CRISPR DESIGN, http://crispr.mit.edu), suggesting a high on-target activity. Two Cas9 cleavage sites were separated from the target site by 12 and 10 bases in forward and reverse sgRNAs, respectively. As a template for HR, two complementary ssODNs of 102 bases in length were designed (Fig. 2a); one was sense direction and the other was anti-sense direction. Both ssODNs contain a single nucleotide transition of ACT to ATT at codon 351 resulting in T315I. To avoid re-cutting of repaired target loci, three silent point mutations were additionally introduced (Fig. 2a). One of three silent point mutations was designed to induce a new EcoRI site (gagttc > gaattc).

### Introduction of T315I mutation in K567

Among four Ph+ myeloid cell lines, we first used K562, which is the most commonly used Ph+ myeloid cell line for the study of TKI sensitivity. To increase HR efficiency, we treated K562 cells with SCR7 (an inhibitor for a DNA ligase IV that is a key enzyme for NHEJ)^33,34^ before and during treatment of the CRISPR/Cas9. We electroporated recombinant Cas9 protein in combination with either forward or reverse sgRNAs and either sense or anti-sense ssODNs. Forty-eight hours after transfection (Fig. 2b), cells were plated into 10 wells of 24-well plate and cultured for 5 more days in the absence of imatinib. Subsequently, the cells were cultured in the presence of 1 μM of imatinib. When the imatinb-resistant cells were selectively expanded, the cells were transferred to culture flask and expanded in the absence of imatinib for further experiments. Imatinib-resistant sublines were obtained in all four combinations of sgRNAs and ssODNs up to day 26 of the imatinib selection (Fig. 2c), while no imatinib-resistant cells were expanded in parental cells during imatinib selection for 30 days (data not shown). Among the four combinations, the combination of reverse sgRNA and anti-sense ssODN revealed significantly higher efficiency than the three other combinations (p < 0.001 in Kaplan-Meier test). To verify whether imatinib-resistant sublines acquired the T315I mutation as a result of HR-mediated gene editing, we extracted genomic DNA, amplified the 427 bp fragment of the *ABL1* gene containing exon 6 by PCR using primers in introns 5 and 6, and subsequently tested EcoRI digestion of each PCR product (Fig. 2d). PCR products of all seven sublines tested were partially digested with EcoRI, whereas that of parental cells was not. Direct sequencing (Fig. 2e) confirmed mixture of T315I and additional silence point mutations with wild-type sequence in each imatinib-resistant subline, indicating that imatinib-resistant sublines acquired the T315I mutation as a result of HR-mediated gene editing with the CRISPR/Cas9 system.

### Significance of SCR7 treatment in the HR efficiency

Next, we verified the significance of SCR7^33,34^ treatment in HR efficiency. We electroporated recombinant Cas9 protein in combination with reverse sgRNA and anti-sense ssODN (which showed the highest HR efficiency in the above analyses) into K562 cells with or without SCR7 treatment. We also electroporated recombinant Cas9 protein in combination with reverse sgRNA and carrier ssODN (which is not template for HR but enables to induce similar transfection efficiency) into K562 cells treated with SCR7. Electroporated cells were cultured in the same way as shown in Fig. 2b. Imatinib-resistant sublines were obtained relatively faster in the cells treated with SCR7 (Fig. 2f) (up to day 17 of imatinib selection) than the untreated cells (up to day 22), although no statistically significant difference was observed between the two groups (p = 0.198 in Kaplan-Meier test). Of note, no imatinib-resistant subline was obtained without specific template ssODN during imatinib selection over 30 days.

### Induction of T315I mutation in TCCS and KOPM28 using the CRISPR/Cas9 system

**Figure 3:**
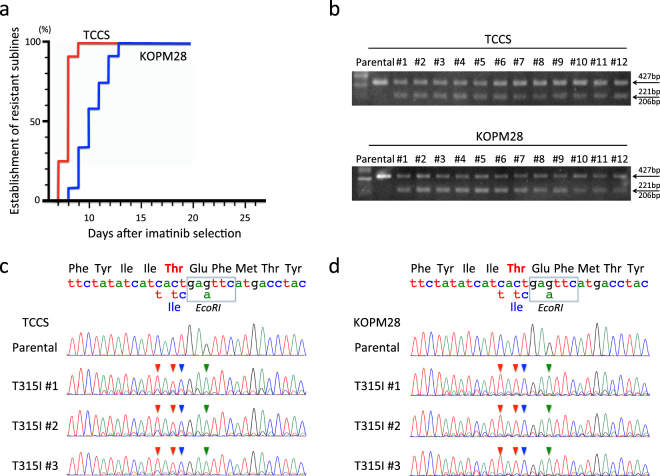
Induction of the T315I mutation in TCCS and KOPM28. (**a**) Efficiencies in establishment of imatinib-resistant sublines. The vertical axis indicates percentage (%) establishment of imatinib-resistant sublines, and the horizontal axis indicates days after imatinib selection. Efficiencies in SCR7-treated TCCS (red) and KOPM28 (blue) electroporated with reverse sgRNA and anti-sense ccODN are indicated. (**b**) Electrophoresis of PCR products after EcoRI digestion. Upper and lower panels indicate parental cells and imatinib-resistant sublines of TCCS and KOPM28, respectively. Full-length gels are presented in Supplementary Figs S2 and S3. (**c**,**d**) Sequences of PCR products. Sequences of parental cells and three imatinib-sublines of TCCS (**c**) and KOPM28 (**d**) are indicated. Wild-type genomic and amino acid sequences and mutations in template sequence for HR are indicated at the top of the panel. Arrowheads in each sequence indicate mutations as a result of HR-mediated gene editing.

Next, we introduced the T315I mutation into two other Ph+ myeloid cell lines (TCCS and KOPM28) resistant to olaparib (Fig. 1). We electroporated recombinant Cas9 protein in combination with reverse sgRNA and anti-sense ssODN into two cell lines treated with SCF7. The electroporated cells were cultured in the same way as shown in Fig. 2b. Imatinib-resistant sublines were efficiently obtained (Fig. 3a) in both TCCS (up to day 9 of imatinib selection) and KOPM28 (up to day 13). Parental cells of TCCS and KOPM28 were completely killed during 14 days culture in the presence of 1 μM imatinib (data not shown). PCR products of genomic DNA from 12 imatinib-resistant sublines of TCCS and KOPM28 were partially digested with EcoRI, unlike those from parental cells (Fig. 3b). Direct sequence of the PCR products from three representative sublines of TCCS (Fig. 3c) and KOPM28 (Fig. 3d) revealed T315I and additional silence point mutations as the main signal, indicating that imatinib-resistant sublines acquired the T315I mutation as a result of HR-mediated gene editing.

### No sign of additional mechanisms for imatinib-resistance in T315I-acquired sublines

**Figure 4:**
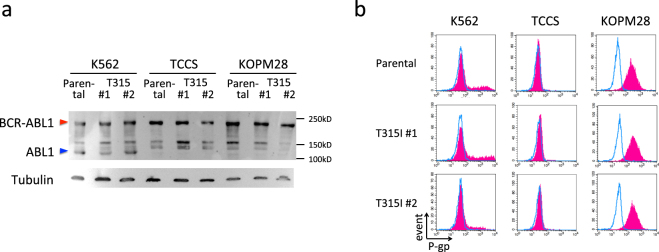
Protein expression of BCR-ABL1 and cell surface expression of P-gp. (**a**) Western blot analysis of BCR-ABL1 in parental cells and imatinib-resistant sublines (T315I #1 and #2) of K562, TCCS, and KOPM28. Upper and lower panels indicate the blotting with anti-ABL and anti-Tubulin antibodies, respectively. Full-length blots are presented in Supplementary Figs S4 and S5. (**b**) Cell surface expression of P-gp in parental cells and imatinib-resistant sublines (T315I #1 and #2) of K562, TCCS, and KOPM28. Blue line and red shade indicate the fluorescence intensities of isotype control and anti-P-gp antibodies, respectively.

Upregulation of BCR-ABL1 protein due to amplification of the *BCR-ABL1* fusion gene^18^ and overexpression of P-gp^19^ were observed in the imatinib-resistant sublines established after long-term culture of imatinib-sensitive Ph+ leukemia cell lines in the presence of increasing concentrations of imatinib. Thus, T315I sublines of three cell lines may acquire these additional mechanisms for imatinib-resistance during imatinib selection. To rule out these possibilities, we evaluated BCR-ABL1 protein expression level in parental cells and imatinib-resistant sublines of three cell lines by Western blotting (Fig. 4a). BCR-ABL1 protein expression levels were similar in both parental cells and imatinib-resistant sublines (T315I #1 and #2) of three cell lines. Thus, amplification of the *BCR-ABL1* fusion gene is unlikely to be associated with imatinib-resistant phenotype.

Next, we analyzed cell surface expression level of P-gp by flow cytometry (Fig. 4b). P-gp expression in three parental cell lines showed different patterns: K562 showed two peaks of major negative and minor positive populations, while TCCS and KOPM28 showed single negative and single positive population, respectively. The expression patterns of P-gp in imatinib-resistant sublines of three cell lines (T315I #1 and #2) were identical to their parental cells thus demonstrating that upregulation of P-gp was not associated with the imatinib-resistant phenotype.

### Acquisition of T315I mutation in *ABL1* transcripts of imatinib-resistant sublines

**Figure 5:**
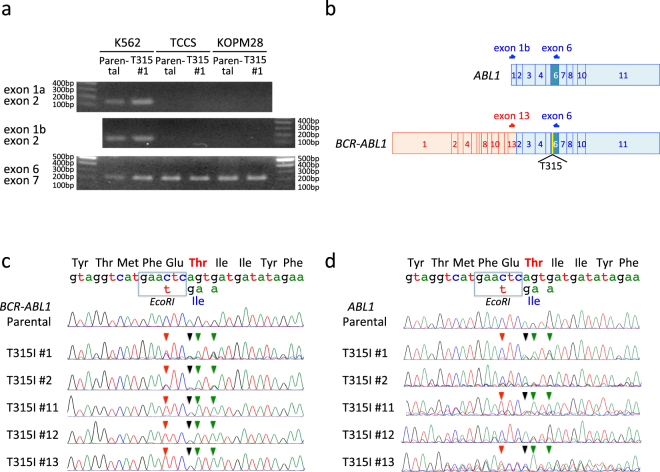
Distinctive evaluation of *ABL1* and *BCR-ABL1* transcripts in K562. (**a**) RT-PCR of *ABL1* and *BCR-ABL1* in parental cells and imatinib-resistant subline (T315I #1) of K562, TCCS, and KOPM28. Upper, middle, and lower panel indicate RT-PCR products of exons 1a and 2, exons 1b and 2, and exons 6 and 7, respectively. Full-length gels are presented in Supplementary Figs S6 and S7. (**b**) Schematic of *ABL1* and *BCR-ABL1* transcripts. Arrows indicate primer for RT-PCR analyses. (**c**,**d**) Sequences of *ABL1* (**c**) and *BCR-ABL1* (**d**) transcripts in parental cells and imatinib-resistant sublines of K562. Direct sequence of RT-PCR products by the reverse primer is indicated. Wild-type mRNA and amino acid sequences in the revers direction and mutations in template for HR are indicated at the top of the panels. Arrowheads in each sequence indicate mutations as a result of HR-mediated gene editing.

In western blotting analysis (Fig. 4a), ABL1 protein was detectable in K562 but not in TCCS and KOPM28. *ABL1* gene consists of 11 exons with alternative splicing exons of 1a and 1b^35^. In the p210 type of *BCR-ABL1* fusion, exons 2–11 of the *ABL1* gene are fused to exons 1–13 of the *BCR* gene^35^. Thus, exons 1a and 1b of the *ABL1* gene are specific for *ABL1* gene, while exons 2–11 of the *ABL1* gene are present in both *ABL1* and *BCR-ABL1* transcripts. Consistent with protein expression, *ABL1*-specific RT-PCR products with primers in exons 1a or 1b and 2 of the *ABL1* gene were detectable in K562 but not in TCCS and KOPM28 (Fig. 5a). In contrast, RT-PCR products with primers in exons 6 and 7 were detectable in three cell lines. These observations demonstrated that K562 has *ABL1* and *BCR-ABL1* alleles whereas TCCS and KOPM28 have only the *BCR-ABL1* allele. Thus, *ABL1* allele is another target of the CRISPR/Cas9 system in K562. To distinctively evaluate the *ABL1* and *BCR-ABL1* alleles in K562 cells, we performed RT-PCR analyses using two sets of primers (Fig. 5b). Primers in exons 1a/b and 6 of the *ABL1* gene were specific for *ABL1* transcripts, while those in exon 13 of the *BCR* gene and exon 6 of the *ABL1* gene were specific for *BCR-ABL1* transcripts. We directly sequenced the RT-PCR products of parental cells as well as five imatinib-resistant sublines of K562 with the reverse primer (Fig. 5b). *BCR-ABL1* transcripts showed two patterns; sublines #1 and #2 revealed mixed pattern of native and HR sequences, while sublines #11, #12, and #13 predominantly showed HR sequence. Taken together, all five sublines had T315I mutation of the *BCR-ABL1* transcripts as a result of HR-mediated gene editing. *ABL1* transcripts in imatinib-resistant sublines showed diverse patterns (Fig. 5c); subline #1 revealed mixed pattern of native and HR sequences, subline #2 mainly revealed native sequence, subline #11 mainly revealed HR sequence, subline #12 revealed mixed pattern of native sequence and one base deletion due to NHEJ, and subline #13 revealed mixed pattern of one base insertion due to NHEJ and HR sequence. Taken together, T315I mutation due to HR-mediated gene editing was detectable in three of five sublines.

### Imatinib-resistance in T315I-acquired sublines

**Figure 6:**
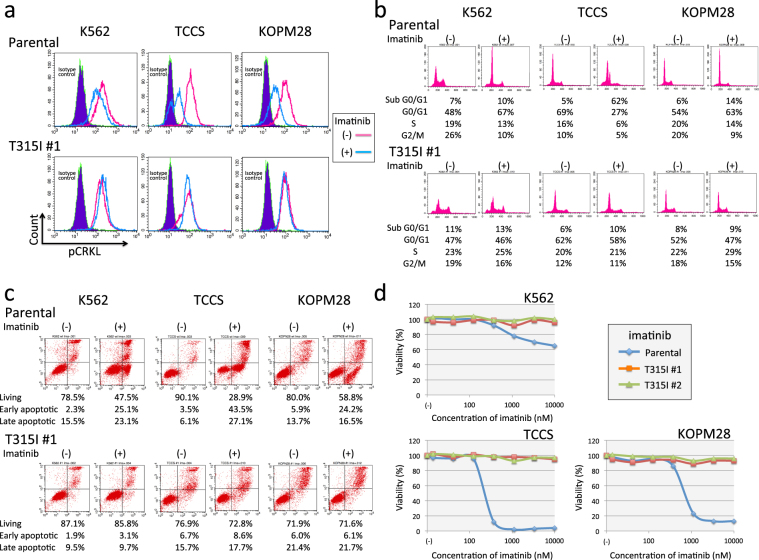
Imatinib sensitivity in parental cells and T315I-acquired sublines of three cell lines. (**a**) Phosphorylation of CRKL in parental cells (upper panel) and T315I-acquired subline (#1) (lower panel) of K562 (left), TCCS (middle), and KOPM28 (right). Cells were cultured in the absence or presence of 1 μM for 24 hours. Blue shade, red line, and blue line indicate isotype control, phospho-CRKL of untreated cells, and phospho-CRKL of imatinib-treated cells, respectively. (**b**) Flow cytometric analysis of PI staining in parental cells (upper) and T315I-acquired subline (#1) (lower panel) of K562 (left), TCCS (middle), and KOPM28 (right). PI staining was performed after 24 hours culture in the absence (upper) or presence (lower) of 1 μM of imatinib. Percentages of cells in sub-G0/G1, G0/G1, S, and G2/M phases are indicated in each panel. (**c**) Flow cytometric analysis of apoptotic cell death in parental cells (upper) and T315I-acquired subline (#1) (lower panel) of K562 (left), TCCS (middle), and KOPM28 (right). Cells were cultured in the absence or presence of 1 μM for 24 (TCCS and KOPM28) or 60 (K562) hours, and analyzed with Annexin V-binding (horizontal axis) and 7AAD-staining (vertical axis) using flow cytometry. The percentages of living cells and early and late apoptotic cells are indicated. (**d**) Dose-response curve of imatinib sensitivity in parental cells (blue line) and T315I-acquired sublines (#1 and #2) (red and green lines, respectively) of K562 (upper left), TCCS (upper right), and KOPM28 (lower left). The vertical axis indicates % viability in the alamarBlue cell viability assay and the horizontal axis indicates log concentration of imatinib (nM).

To precisely confirm the effect of the T315I mutation, we first analyzed the phosphorylation status of CRKL (one of critical downstream key enzymes of BCR-ABL1)^36,37^ using flow cytometry (Fig. 6a). In the parental cells of three cell lines, CRKL was dephosphorylated by imatinib treatment. In contrast, in the T315I-acquired subline of three cell lines, CRKL was constitutively phosphorylated, even after imatinib treatment. Next, we analyzed the induction of cell cycle arrest (Fig. 6b) and apoptotic cell death (Fig. 6c) by imatinib using flow cytometry. Imatinib treatment of parental cells induced accumulation into the G0/G1 phase in K562 and KOPM28. Furthermore, in parental cells of TCCS, over half of the treated cells accumulated into the sub-G0/G1 phase. In contrast, in T315I-acquired sublines of three cell lines, cell cycle arrest was not induced by imatinib treatment. Furthermore, imatinib treatment induced apoptotic cell death in parental cells, but not in the T315I-acquired sublines of three cell lines. We finally determined dose-response curves of imatinib using the alamarBlue cell viability assay (Fig. 6d). T315I-acquired sublines of three cell lines showed a marked resistance to imatinib (up to 10 μM).

### Drug sensitivities of T315I-acquired sublines

**Figure 7:**
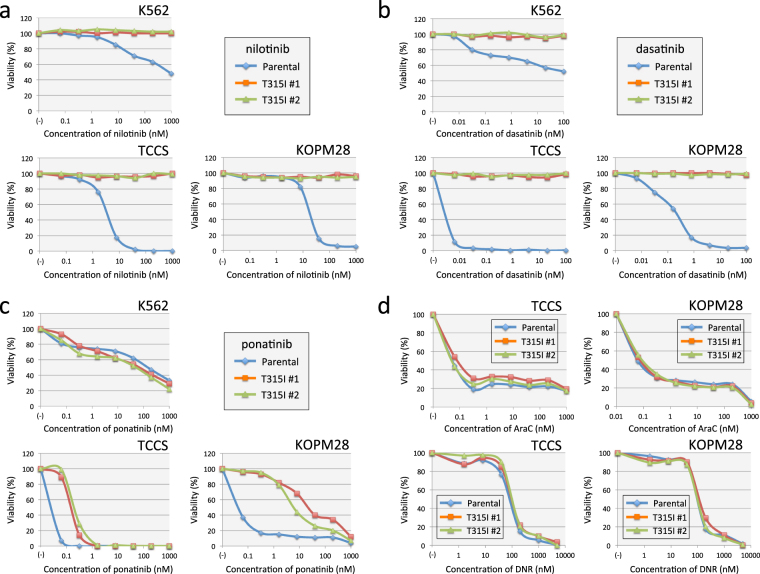
TKIs and chemotherapeutic agents sensitivities in parental cells and T315I-acquired sublines of three cell lines. (**a**,**b**,**c**) Dose-response curves of nilotinib (**a**), dasatinib (**b**), and ponatinib (**c**) sensitivities in parental cells (blue line) and T315I-acquired sublines (#1 and #2) (red and green lines, respectively) of K562 (upper left), TCCS (upper right), and KOPM28 (lower left). The vertical axis indicates % viability in the alamarBlue cell viability assay and the horizontal axis indicates log concentrations of nilotinib (**a**), dasatinib (**b**), and ponatinib (**c**) (nM). (**d**) Dose-response curves of AraC (upper) and DNR (lower panel) sensitivities in parental cells (blue line) and T315I-acquired sublines (#1 and #2) (red and green lines, respectively) of TCCS (left) and KOPM28 (right panel). The vertical axis indicates % viability in the alamarBlue cell viability assay and the horizontal axis indicates log concentrations of AraC and DNR.

We evaluated the sensitivities to second-generation TKIs using the alamarBlue cell viability assays. T315I-acquired sublines of three cell lines showed marked resistance to nilotinib (Fig. 7a) and dasatinib (Fig. 7b) —up to 1 μM and 100 nM, respectively. Next, we evaluated the sensitivity to ponatinib (Fig. 7c). Parental cells and T315I-acquired sublines of K562 showed similar dose-response curves, and their IC_50_ values were approximately 100 nM, which was lower than mean peak concentration (137 nM) of orally administrated ponatinib (45 mg daily) at steady state^38^. Parental cells of TCCS and KOPM28 were highly sensitive to ponatinib and their IC_50_ values were less than 0.1 nM. T315I sublines of TCCS and KOPM28 were significantly more resistant to ponatinib than their parental cells; their IC_50_ values were approximately 0.2 nM and 20 nM, respectively. We also analyzed the sensitivities to chemotherapeutic agents, AraC and DNR (Fig. 7d) except for K562, which were highly resistant to these agents (data not shown). Parental cells and T315I-acquired sublines of TCCS and KOPM28 were equally sensitive to AraC and DNR, indicating that the T315I mutation of BCR-ABL1 specifically induced resistance to TKI but not to chemotherapeutic agents.

## Discussion

In the present study, we tried to introduce the T315I mutation of *BCR-ABL1* into Ph+ leukemia cell lines by HR-mediated gene editing with the CRISPR/Cas9 system. Since BCR-ABL1 has been reported to repress a variety of genes involved in the HR pathway^24–28^, we analyzed their sensitivities to olaparib (a PARP1 inhibitor) to functionally evaluate the status of the HR pathway. We found that all four Ph+ myeloid leukemia cell lines were highly resistant, suggesting that their HR pathway is functionally not impaired. Indeed, we successfully established imatinib-resistant sublines of three Ph+ myeloid cell lines—K562, TCCS, and KOPM28—which acquired the T315I mutation as a result of HR-mediated gene editing. We previously tried to introduce specific gene mutations of our interested genes in four Ph− ALL cell lines with the same strategy, but we failed to obtain sublines with the desired mutation. Of note, in the present study, all of the four cell lines that we used in the previous attempts turned out to be sensitive to PARP-1 inhibitor, suggesting that their HR pathway was functionally impaired. Thus, although further verifications are required, sensitivity of cancer cell lines to PARP1 inhibitor may be a useful biomarker to predict efficiency of HR-mediated gene editing with the CRISPR/Cas9 system.

To select successfully integrated cells, we used imatinib. In the previous reports^15–17^, imatinib-resistant sublines with T315I were established from Ph+ leukemia cell lines after 5–10 months’ culturing in the presence of increasing concentrations of imatinib. Long-term culture in the presence of increasing concentrations of imatinib also induced enhancement of BCR-ABL1 expression^18^ and/or upregulation of cell surface expression of P-gp^19^ as mechanisms for imatinib-resistance, suggesting that T315I-positive sublines of Ph+ leukemia cell lines may acquire additional mechanism(s) for imatinib-resistance during imatinib selection. Considering these previous reports, to allow an expansion of T315I integrated cells and prevent a spontaneous emergence of imatinib-resistant sublines, we initially cultured cells for 7 days in the absence of imatinib after electroporation. Furthermore, we used 1 μM of imatinib for selection to eliminate emergence of clones that are moderately resistant to imatinib due to mechanism(s) other than an acquisition of T315I mutation. As a result, we successfully expanded T315I acquired sublines of three myeloid Ph+ leukemia cell lines after short-term (7 to 26 days) imatinib selection. Enhancement of BCR-ABL1 expression and upregulation of cell surface expression of P-gp were not induced in imatinib-resistant sublines of three cell lines.

We prepared forward and reverse sgRNAs in combination with sense and anti-sense ssODNs. Efficiency of HR-mediated gene editing is reported to be affected by structure and composition of sgRNA and ssODN such as incorporation of silent mutation to block re-cutting of repaired loci^39^, distance between mutation site and cleavage site^39^, length of homology arms^40,41^, and strand complementarity or orientation of ssODN^40–42^. In the present study, all four combinations produced identical distance between mutation site and cleavage site and introduced three silent mutations to block re-cutting of repaired loci. The highest efficiency was observed in the combination of reverse sgRNA and anti-sense ssODN, which was complementary to the transcribed strand and contained the NGG PAM sequence. This was inconsistent with previous reports using different delivery and modality of CRISPR/Cas9 system, since higher efficiency was reported to be obtained by ssODN that was complementary to the non-transcribed strand^42^ and by ssODN that was complementary to the PAM strand^41^. Thus, delivery and modality of CRISPR/Cas9 system might affect an optimal combination of sgRNA and ssODN.

TCCS and KOPM28 have the *BCR-ABL1* allele but not the *ABL1* allele. We confirmed that all of the imatinib-resistant sublines of two cell lines had both native and recombination alleles of *BCR-ABL1*, suggesting that these two cell lines may serve as good models of acquired T315I mutation in BCR-ABL1. In contrast to TCCS and KOPM28, K562 has both *BCR-ABL1* and *ABL1* alleles, and its *ABL1* allele also acquired the T315I mutation. Although it is unlikely that the induced ABL1 T315I mutation contributes to imatinib resistance, this may be a confounding issue limiting the use of the K562 subclones for drug testing. Removal of the *ABL1* allele by a CRISPR/Cas9-induced deletion in K562 may resolve this problem.

T315I sublines from the three cell lines showed a marked resistance to dasatinib and nilotinib, as well as imatinib in comparison with their parental cells. Although ponatinib was developed as a potent TKI that can inhibit all critical kinase domain mutations including T315I, its activity against the T315I mutant was reported to be modestly decreased in comparison with the activity against native ABL1 and BCR-ABL1 as follows^10^. The IC_50_ value of ponatinib in the kinase assay *in vitro* was reported to be 0.37 and 2 nM for native ABL1 and T315I mutant, respectively. In Baf3 transfectants, the IC_50_ value of ponatinib was reported to be 0.5 and 11 nM for native BCR-ABL1 and T315I mutant, respectively^10^. In the present study, T315I sublines of TCCS and KOPM28 were sensitive to ponatinib, and their IC_50_ values (approximately 0.2 and 20 nM, respectively) were significantly higher than those in parental cells (less than 0.1 nM). In K562, the sensitivities of parental cells and T315I sublines were identical. These observations demonstrated that parental cells and T315I sublines of three cell lines are useful models for the analysis of TKI sensitivities.

Brabetz *et al*.^43^ recently introduced an oncogenic mutation (R140Q) of the *IDH2* gene into K562 cells successfully using the CRISPR/Cas9 system. Since R140Q is located in exon 4 of the *IDH2* gene, they inserted exons 4–11 with R140Q as a partial cDNA into exon 4 of the *IDH2* gene locus via HR. Thus, the endogenous *IDH2* gene promoter regulated the integrated *IDH2* R140Q mutation, but the insertion lacks introns and 3′-untranslated regions (UTRs) in the target gene. Recently, it has been reported that introns^44^ and 3′UTRs^45,46^ are involved in transcriptional and post-transcriptional regulation of gene expression. Indeed, 3′UTR of the *BCR-ABL1* transcripts were reported to be target of microRNA-203 (miR-203)^47^, miR-424^48^ and miR-30e^49^. In the present study, the introduction of a precise mutation into the *BCR-ABL1* gene by harnessing the cells’ HR repair pathway enabled preservation of other regulatory regions in the gene and minimized off-target effects relative to the more disruptive changes induced by NHEJ driven repair.

## Methods

### Cell lines

Twenty Ph-positive (Ph+) leukemia cell lines (16 lymphoid and four myeloid) listed in Table 1 were analyzed. K562, TCCS, KOPM28, and KOPM30 were Ph+ myeloid cell lines. KOPN30bi, KOPN56, KOPN57bi, KOPN66bi, KOPN72bi, KOPN83bi, YAMN73, YAMN91, Kasumi8, Nalm1, Nalm27, KCB1, TCCY, SU-Ph2, and SK9 were Ph+ B-cell precursor leukemia cell lines. K562, TCCS, KOPM28, KOPN55bi, and Nalm1 were established from CML-BC with the p210 BCR-ABL1. KOPM30 and KOPN30bi were established from the same patient with Ph+ acute leukemia with the p190 BCR-ABL1 at different stages of the disease. KOPN56, TCCY, and Nalm27 were established from Ph+ acute lymphoblastic leukemia (ALL) with the p210 BCR-ABL1. YAMN73 was established from Ph+ ALL with the p203 BCR-ABL1 rearranged in major *BCR*. KOPN57bi, KOPN66bi, KOPN72bi, KOPN83bi, YAMN91, Kasumi8, KCB1, SU-Ph2, and SK9 were established from Ph+ ALL with the p190 BCR-ABL1. Seventy-seven Ph- BCP-ALL cell lines listed in Supplemental Table 1 were used as control. All cell lines were maintained in RPMI1640 medium supplemented with 10% fetal calf serum (FCS).

### alamarBlue cell viability assay

To determine the sensitivities to olaparib, imatinib, dasatinib, nilotinib, ponatinib, cytosine arabinoside (AraC), and daunorubicin (DNR), an alamarBlue cell viability assay (Bio-Rad Laboratories, Hercules, CA) was performed. A total of 1–5 × 10^5^ cells were plated into a 96-well flat-bottom plate in triplicate and cultured for 68 hours in the presence or absence of seven concentrations of each drug. After a 6 hour additional incubation with alamarBlue, absorbance at 570 nm were monitored by a microplate spectrophotometer using 600 nm as a reference wavelength. Cell survival was calculated by expressing the ratio of the optical density of treated wells to that of untreated wells as a percentage. The concentration of drug required to reduce the viability of treated cells to 50% of untreated cells was calculated, and the median of three independent assays was determined as 50% inhibitory concentration (IC_50_) for each cell line.

### Introduction of the T315I mutation by the CRISPR/Cas9 system

Two CRISPR guide sequences were designed using the CRISPR design tool (CRISPR DESIGN, http://crispr.mit.edu) as follow; 5′-atcactgagttcatgacctacgg-3′ (forward sgRNA) and 5′-aactcagtgatgatatagaacgg-3′ (reverse sgRNA). 102 base pair template ssODN in a transcribed strand (sense) direction and its complementary template ssODN in a non-transcribed strand (anti-sense) direction were synthesized by Integrated DNA technologies (Coralville, IA, USA). The sequence of ssODN in the sense direction was as follows; 5′-gggtctgcacccgggagcccccgttctatatcatTaTCgaAttcatgacctacgggaacctcctggactacctgagggagtgcaaccggcaggaggtgaacg-3′. Four mutated nucleotides are capitalized. As a negative control, we used carrier ssODN (Integrated DNA technologies). Recombinant Cas9 nuclease with guide RNA (Integrated DNA technologies) was electroporated into cell lines as a ribonucleoprotein (RNP) complex with template ssODN following manufacturer’s protocol^50^. Briefly, RNP complex and template ssODN were electroporated into 1 × 10^5^ cells, which were untreated or pretreated with 10 nM of SCR7^33,34^ (Cayman Chemical, Ann Arbor, MI, USA) for 24 hours before electroporation, using Neon electroporation transfection system (Thermo Fisher Scientific). The electroporated cells were cultured in the absence or presence of 10 nM of SCR7 for 24 hours in one well of 96 well plate. Forty-eight hours after electroporation, the electroporated cells were mixed with untreated cells and plated into 10–12 wells of 24-well plate. After 5 days expansion in the absence of imatinib, the cells were cultured in the presence of 1 μM of imatinib. During imatinib selection, half of the culture medium in each well was exchanged every 2–3 day with fresh medium containing 1 μM of imatinib. When the imatinb-resistant cells were selectively expanded, imatinb-resistant sublines in each well were transferred to culture flask and expanded in the absence of imatinib for further experiments.

### PCR analyses

To confirm HR in the *BCR-ABL1* fusion gene, genomic DNA samples extracted from parental and imatinib-resistant sublines were amplified by PCR using forward (5′-ccacacgagcacagtctcag-3′) and reverse (5′-cctaggctggggctttttgta-3′) primers that were specific for introns 5 and 6 of the *ABL1* gene, respectively. Each PCR product digested with EcoRI was electroporated. Direct sequencing of each PCR product was performed using the forward primer. To detect *ABL1* transcripts, cDNA prepared from parental and imatinib-resistant sublines was amplified by RT-PCR using forward primers in exons 1a (5′-gtgggctgcaaatccaagaag-3′) or 1b (5′-aggaatcatcgaggcatggg-3′) and reverse primer in exon 2 (5′-gtccagcgagaaggttttcc-3′). As a control, RT-PCR was also performed using forward primer in exons 6 (5′-ctgctgtacatggccactca-3′) and reverse primer in exon 7 (5′-ctctcgggtgcagtccattt-3′). To distinctively sequence the *ABL1* and the *BCR-ABL1* transcripts, RT-PCR was performed using forward primers in exon 1b of the *ABL1* gene (5′-ccacactgcaatgtttttgtgg-3′) or exon 13 of the *BCR* gene (5′-agcattccgctgaccatcaa-3′) and reverse primer in exon 6 of the *ABL1* gene (5′-tctcccctaccaggcagttt-3′), respectively, and direct sequencing of the RT-PCR products was performed using the reverse primer.

### Flow cytometric analyses

To evaluate cell surface expression of P-glycoprotein, cells were stained with a fluorescein isothiocyanate (FITC)-conjugated anti-P-glycoprotein antibody (Nichirei, Tokyo, Japan) and analyzed by flow cytometry (FACSCalibur, BD Biosciences, San Jose, CA). To analyze phosphorylation status of CRKL, cell cycle, and apoptotic cell death, cells were cultured in the presence or absence of 1 μM of imatinib for 24 hours except for detection of apoptosis in K562 cells (60 hours incubation). To evaluate phosphorylation status of CRKL, the cells were fixed with 10% formaldehyde at 37 °C for 10 min and permeabilized with 90% methanol on ice for 30 min. After washing the cells twice with PBS/0.5% BSA, the cells were stained with anti-phospho CRKL (pTyr207) (#3811, Cell Signaling Technology, Danvers, MA, USA) and subsequently with Alexa Fluor 488-conjugated goat anti-rabbit IgG (H + L) (#A11008, Invitrogen, Carlsbad, CA, USA) and analyzed by flow cytometry. For cell cycle analysis, the cells fixed with 70% ethanol were stained with propidium iodide (PI) (Sigma, St. Louis, MO) and analyzed by flow cytometry. To detect apoptosis, the cells were stained with FITC-conjugated Annexin-V and 7AAD (MBL, Nagoya, Japan) and analyzed by flow cytometry.

### Western blot analyses

Cells were solubilized in lysis buffer (50 mM Tris-HCl, pH 7.5, 150 mM NaCl, 1% Nonidet P-40, 5 mM EDTA, 0.05% NaN_3_, 1 mM phenylmethylsulfonyl fluoride, 100 μM sodium vanadate). The lysates were separated on a SDS-polyacrylamide gel under reducing conditions and then transferred to a nitrocellulose membrane. The membrane was incubated with anti-ABL1 antibody (#554148, BD Biosciences, Franklin Lakes, NJ, USA) and anti-alpha-Tubulin antibody (T5168, Sigma) at 4 °C overnight, and then with horseradish peroxidase-labeled second antibody (MBL, Nagoya, Japan) at room temperature for 1 hour. The bands were developed using an enhanced chemiluminescence detection (ECL) kit (GE Healthcare, Little Chalfont, UK).

### Statistics

Mann-Whitney test was applied for comparison of drug sensitivities, a paired t-test was for comparison in the same subject, and Kaplan-Meier analysis for comparison of efficiencies in the establishment of imatinib-resistant sublines.

### Data availability

The authors declare that the data supporting the findings of this study are available within the paper and its supplementary information files.

## Electronic supplementary material

Supplementary Figure S1–7
