# Supplementary material for: T315I mutation of BCR-ABL1 into human Philadelphia chromosome-positive leukemia cell lines by homologous recombination using the CRISPR/Cas9 system
Source: Sci Rep. 2018 Jul 2;8:9966. doi: 10.1038/s41598-018-27767-6 (PMC6028382; doi:10.1038/s41598-018-27767-6)

**T315I mutation of BCR-ABL1 into human Philadelphia chromosome-positive leukemia cell lines by homologous recombination using the CRISPR/Cas9 system**

Minori Tamai^1^, Takeshi Inukai^1^, Satoru Kojika^1^, Masako Abe^1^, Keiko Kagami^1^, Daisuke Harama^1^, Tamao Shinohara^1^, Atsushi Watanabe^1^, Hiroko Oshiro^1^, Koshi Akahane^1^, Kumiko Goi^1^, Eiji Sugihara^2^, Shinichiro Nakada^3^, Kanji Sugita^1^

1. Department of Pediatrics, School of Medicine, University of Yamanashi, Chuo, Japan
2. Innovation Medical Research Institute, University of Tsukuba, Ibaraki, Japan
3. Department of Bioregulation and Cellular Response, Graduate School of Medicine, Osaka University, Osaka, Japan

Supplementary Figure S1. Full‐length gel of representative image for Fig. 2d.


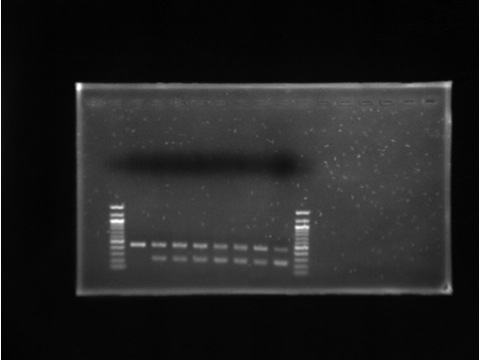


Supplementary Figure S2. Full‐length gel of representative image for Fig. 3b upper panel.


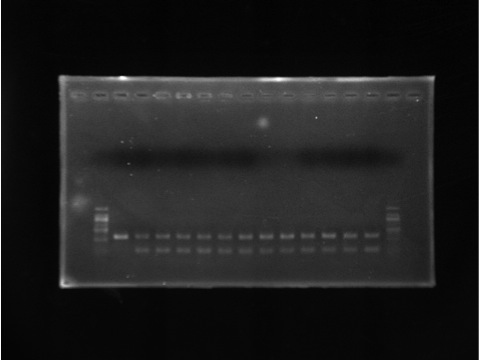


Supplementary Figure S3. Full‐length gel of representative image for Fig. 3b lower panel.


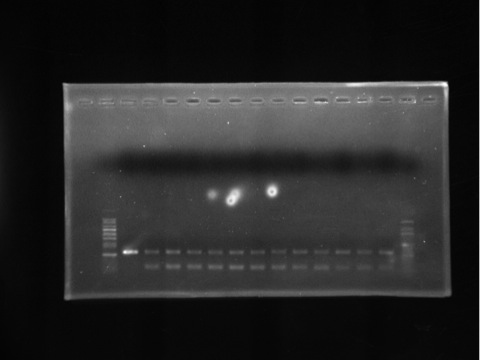


Supplementary Figure S4. Full‐length blot of representative image for Fig. 4a upper panel.


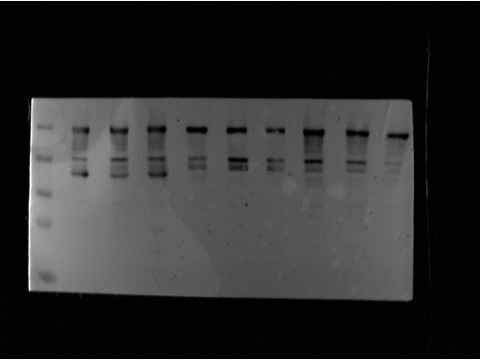


Supplementary Figure S5. Full‐length blot of representative image for Fig. 4a lower panel.


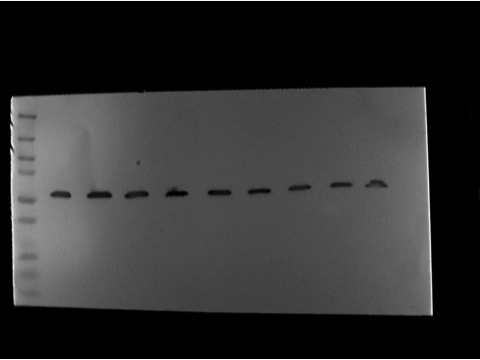


Supplementary Figure S6. Full‐length gel of representative image for Fig. 5a upper and middle panels.


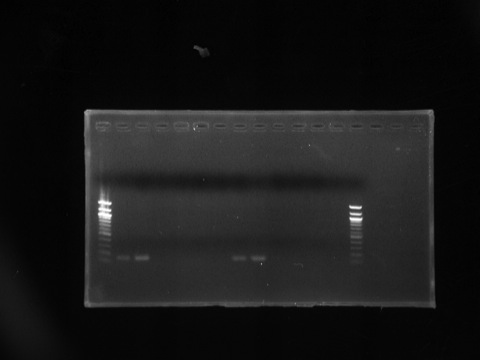


Supplementary Figure S7. Full‐length gel of representative image for Fig. 5a lower panel.


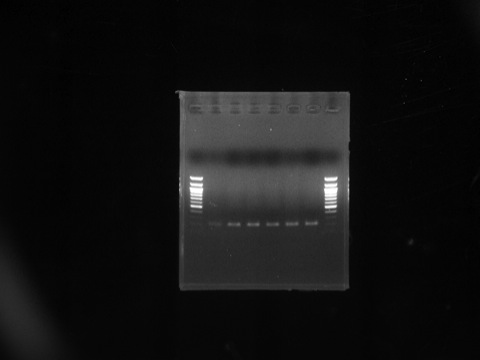

Supplement: Supplementary file 1 — Supplementary Figure S1–7 [file 41598_2018_27767_MOESM1_ESM.docx]
